# Commentary: Statistical Adhockeries Are No Criteria for Legal Decisions—The Case of the Expert Medical Report on the Assessment of Urine Specimens Collected Among Athletes Having Participated to the Vancouver and Sochi Winter Olympic Games

**DOI:** 10.3389/fsoc.2019.00085

**Published:** 2019-12-20

**Authors:** Michel Burnier

**Affiliations:** Faculty of Biology and Medicine, University of Lausanne, Lausanne, Switzerland

**Keywords:** expertise, statistical approach, plausibility, Bayesian (subjective) probability, coherence

As cited by Gelman and Hennig, “*Decisions in statistical data analysis are often justified, criticized or avoided by using concepts of objectivity and subjectivity*” (Gelman and Hennig, 2017).

The paper by Taroni et al. (2018), that I have read with interest and some surprise, does not escape this principle. Indeed, it heavily and namely criticizes the statistical approach that was used in a recent expertise that I performed on behalf of the Medical and Scientific Department of the International Olympic Committee (Taroni et al., 2018). The authors indirectly suggest that the analysis could provide “*erroneous conclusions in legal proceedings risk endangering the fairness of the proceedings and can lead to miscarriages of justice*” (Taroni et al., 2018). I understand the worry of the authors who belong to a School of Criminal Justice, to provide as much as possible reliable and unbiased expert conclusions to assist judiciary in their decision-making processes, and I do have the same preoccupation. I do not want to debate on whether a purely statistical approach is more or less appropriate than another statistical method using a Bayesian approach and the calculation of a probability to make an odd observation. Indeed, it is well possible that a Bayesian approach could be superior and more useful for the judges although this remains to be demonstrated in the particular case. The main reason why I would like to react on the content of Taroni's publication is because it seems obvious that the authors have not read the expertise completely and have not clearly understood its purpose and its analysis. Thus, they have not taken into account what Gelan and Hennig call “*the context dependence*” (Gelman and Hennig, 2017). The first important issue is the question asked to the expert. In this case, the first demand was “to determine reference values for various urinary analytes (sodium, potassium, chloride, calcium, creatinine, and urinary density) coming from samples taken from top athletes tested at the time of Vancouver XXI Winter Olympic Games.” This goal could be achieved only with a statistical approach taking into account the distribution of the values of athletes having participated in the Vancouver Games. The second objective was to examine the distribution and statistics of each sample collected from the XXII Olympic Winter Games, which occurred in Sochi and to evaluate them in the light of the reference values obtained in Vancouver. As Taroni et al. correctly pointed out, the populations were different, the former containing athletes of all countries, including Russia, and the latter only samples from Russia and this might have explained some differences due to country-specific diets. This is reason why two analyses were done, one within each population, and one between populations. With this approach, some values were clearly outside the distribution of both the Vancouver and the Sochi populations of athletes and could be considered as “outliers” or extremes of extremes as Taroni et al. name them.

Of note, our objective was not to determine who was doped or not, identifying the presence of a prohibited substance. The baseline hypothesis was that some samples had been manipulated and urine perhaps reconstituted with an excess of salt to match the initial urinary density that was the only parameter available. Therefore, the expert focused on samples with very high sodium and chloride concentrations, which could fit with the hypothesis. Samples eventually manipulated but with a normal sodium chloride concentration would of course escape from this strategy.

Now, if no Bayesian analysis was performed in this expertise to assess the probabilities of extremes to be real outliers, other aspects of plausibility were considered in my analysis using an approach fitting with the abductive approach discussed recently by Simon and Dettweiler (2019). One of them is the coherence between several measured analytes. Indeed, humans are not eating sodium chloride but a diet containing salt but also potassium and calcium. Consequently, humans on a very high salt diet also ingest more calcium and potassium. Interestingly, in the outliers of the Sochi group of athletes, there was a clear gap between urinary sodium and chloride excretions and the excretion of potassium, this latter being in the normal range and comparable to the athletes tested in Vancouver. To a certain degree, the same was true for calcium. Thus, there appears to be incoherence between the urinary content of analytes in subjects recognized as outliers based on urinary sodium concentrations. In addition, one must also take into account the physiological plausibility when examining samples. In some of the athletes, the measured urinary sodium concentrations were so high that they were incompatible with human physiology and were therefore more than suspect. At last, the level of plausibility became extremely high when one noted that several outliers were not isolated athletes but fellow-members of the same competition team.

Thus, Taroni et al. had the impression that our conclusions were based only on a statistical analysis but this is clearly wrong. Interestingly, in their publication Taroni et al. do not propose any alternative for this kind of analysis and even suggest in their publication that results might be the same using a Bayesian approach. Today, the data are available and the authors are welcome to confront their approach with the one used in my expertise. But, as long as no comparison has been performed, the results of my expertise must be considered as correct and reliable and judges can use these data to integrate them in the overall set of evidence, to make their opinion and finally take their decisions.

## Author Contributions

The author confirms being the sole contributor of this work and has approved it for publication.

### Conflict of Interest

The author declares that the research was conducted in the absence of any commercial or financial relationships that could be construed as a potential conflict of interest.
